# Auranofin-loaded nanoparticles as a new therapeutic tool to fight streptococcal infections

**DOI:** 10.1038/srep19525

**Published:** 2016-01-18

**Authors:** Roberto Díez-Martínez, Esther García-Fernández, Miguel Manzano, Ángel Martínez, Mirian Domenech, María Vallet-Regí, Pedro García

**Affiliations:** 1 Departamento de Microbiología Molecular y Biología de las Infecciones, Centro de Investigaciones Biológicas (CSIC), 28040 Madrid, Spain; 2 CIBER de Enfermedades Respiratorias (CIBERES), Madrid, Spain; 3 Departamento de Química Inorgánica y Bioinorgánica, Facultad de Farmacia, Universidad Complutense de Madrid, Instituto de Investigación Sanitaria Hospital 12 de Octubre, i+12, Pz/ Ramón y Cajal s/n, 28040, Madrid, Spain; 4 CIBER de Bioingeniería, Biomateriales y Nanomedicina (CIBER-BBN), Spain

## Abstract

Drug-loaded nanoparticles (NPs) can improve infection treatment by ensuring drug concentration at the right place within the therapeutic window. Poly(lactic-co-glycolic acid) (PLGA) NPs are able to enhance drug localization in target site and to sustainably release the entrapped molecule, reducing the secondary effects caused by systemic antibiotic administration. We have loaded auranofin, a gold compound traditionally used for treatment of rheumatoid arthritis, into PLGA NPs and their efficiency as antibacterial agent against two Gram-positive pathogens, *Streptococcus pneumoniae* and *Streptococcus pyogenes* was evaluated. Auranofin-PLGA NPs showed a strong bactericidal effect as cultures of multiresistant pneumococcal strains were practically sterilized after 6 h of treatment with such auranofin-NPs at 0.25 μM. Moreover, this potent bactericidal effect was also observed in *S. pneumoniae* and *S. pyogenes* biofilms, where the same concentration of auranofin-NPs was capable of decreasing the bacterial population about 4 logs more than free auranofin. These results were validated using a zebrafish embryo model demonstrating that treatment with auranofin loaded into NPs achieved a noticeable survival against pneumococcal infections. All these approaches displayed a clear superiority of loaded auranofin PLGA nanocarriers compared to free administration of the drug, which supports their potential application for the treatment of streptococcal infections.

Bacterial infections are responsible for significant morbidity and mortality in clinical settings and represent a global health threat and a burden to healthcare systems^1^. *Streptococcus pneumoniae*, the pneumococcus, is a Gram-positive pathogen and a leading cause of diseases such as otitis media, bacteremia and meningitis in young children, the elderly, and persons with chronic medical conditions. Its clinical burden is about 2 million deaths per year from invasive disease (defined as isolation of *S. pneumoniae* from a normally sterile site such as blood or cerebrospinal fluid), half of these in children under 5 years of age, but is likely to cause many more due to non-bacteremic pneumonia and other respiratory diseases^2^. Thus, for every case of bacteremic pneumococcal pneumonia in adults, it has been estimated that there are, at least, 3 additional cases of non-bacteremic pneumococcal pneumonia^3^. The classical treatment to fight pneumococcal infections has been the use of antibiotics, but the effectiveness of this therapy has been compromised by the progressive selection for resistance against major drug classes, and treatment failures are widely reported^4^,^5^. In addition, a smaller, but growing, number of pneumococcal isolates are resistant to multiple antibiotics, leaving vancomycin as a drug of last choice^6^. *Streptococcus pyogenes* is also an important human pathogen being the bacterium most frequently isolated from patients with pharyngitis, although it causes more severe invasive infections, including necrotizing fasciitis, sepsis, and toxic shock syndrome. Antibiotic treatment failures in cases of streptococcal pharyngitis have been reported, mainly due to the biofilm formation^7^. Consequently, in a recent report, the US Centers for Disease Control and Prevention (CDC) has called for an aggressive and immediate action to halt the spread of drug-resistant pathogens^8^.

It is well established that drug discovery and development is nowadays a very expensive, time-consuming, and risky process. The so-called drug ‘repurposing’ (or ‘reprofiling’) is an alternative and promising strategy to speed up this drug discovery process, with a concomitant reduction of failure rates and associated costs^9^,^10^. In this sense, auranofin is a mixed ligand gold compound approved by the U.S. Food and Drug Administration (FDA) in 1985, commercialized under the brand name of Ridaura, and recommended for the treatment of severe rheumatoid arthritis^11^. Several years ago, new attractive pharmaceutical activities were disclosed for auranofin including anticancer, antiviral and against pathogenic protozoa, like *Plasmodium falciparum, Entamoeba histolytica*, and *Giardia lamblia*^12^,^13^. Although the antibacterial activity of auranofin has been less explored, a promising effect on certain Gram-positive pathogens as *Clostridium difficile*^14^, *Staphylococcus aureus*^15^ or *Mycobacterium tuberculosis*^16^ has been reported in the recent years.

One of the most successful strategies for overcoming microbial resistance is the use of nanoparticles (NPs) as drug delivery systems, since they can achieve a predictable and desired therapeutic effect in the human body^17^. This effect is reached when the drug plasma concentration at the relevant site is within the therapeutic window, that is, below the toxic level but above the effective level. Thus, sustained release of antibiotics from NPs could potentially improve the treatment efficacy. Additionally, NPs have been used to target antimicrobial agents to the site of infection, so that higher doses of drug can be given at the infected site, thereby increasing bactericidal activity with fewer adverse side effects^18^. Poly(lactic-co-glycolic acid) (PLGA) is one of the most successfully employed biodegradable polymers because the monomers produced after its hydrolysis, lactic acid and glycolic acid, are endogenous and efficiently metabolized by the body. In fact, from all the possible biomaterials established for the production of NPs, PLGA has engaged the interest of the drug delivery community because of its attractive properties. Among them, it is worth to mention: a) the biodegradability and biocompatibility; b) its capability for protecting the drug molecules from biodegradation; and c) the potential development of sustained release systems^19^,^20^,^21^. PLGA NPs have been employed for vaccine delivery^22^, cancer treatment^23^, treatment of inflammatory diseases^24^,^25^, on regenerative medicine^26^,^27^ and even for the treatment of some cardiovascular diseases^28^. Regarding infection treatments, there are some initial studies aimed at developing antibiotic-PLGA NPs to improve the treatment of certain bacterial infections. Different antibiotics have been encapsulated into PLGA NPs, such as rifampicin and azithromycin^29^, gentamicin^30^,^31^,^32^, nafcillin^33^, and sparfloxacin^34^. After several preclinical evaluations, PLGA NPs have demonstrated their antiinfective potential based upon two principles: the NPs are usually taken up by endocytosis, so antibiotic-PLGA NPs are promising delivery systems for targeting intracellular infections; and NPs can achieve sustained release, so antibiotic-PLGA NPs can be employed for the treatment or prevention of infections.

In this work we have explored the use of PLGA NPs loaded with auranofin to demonstrate, as a proof of concept, their efficiency as antibacterial agent against two important streptococcal pathogens, *i.e., S. pneumoniae* and *S. pyogenes*. Promising results have been obtained in *in vitro* assays using multiresistant pneumococcal strains and a biofilm model of *S. pneumoniae* and *S. pyogenes*, as well as *in vivo* using a zebrafish embryo infection model.

## Results

### Morphology of the NPs

**Figure 1:**
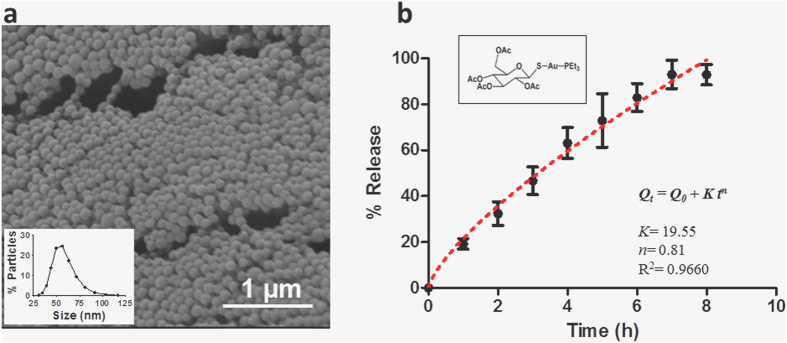
Auranofin and PLGA NPs. (**a**) SEM micrograph of unloaded PLGA NPs and size distribution of PLGA NPs measured by DLS (inset). (**b**) *In vitro* release of auranofin from PLGA NPs and kinetic fitting (dashed line) of the release kinetics according to a near-zero order. Chemical structure of auranofin is inserted in the panel. Ac, acetyl; Et, ethyl.

PLGA NPs, both unloaded and auranofin-loaded, were produced following the nanoprecipitation method described in Methods section. Auranofin was successfully loaded into the PLGA nanocarrier, as digestion experiments confirmed. The morphology of NPs was observed by scanning electron microscopy (SEM) (Fig. 1a), showing the expected spherical shape of the produced NPs. Loading auranofin during the nanoprecipitation stage did not affect the morphology of the particles (data not shown).

### Average diameter and zeta potential

Size of NPs was evaluated using a Dynamic Light Scattering (DLS) apparatus. The mean diameter of both unloaded and auranofin-PLGA NPs was observed to be *ca.* 60 nm in both cases (Fig. 1a). The surface characteristics were examined through zeta potential, and all NPs were negatively charged with values *ca.* −30 mV, as expected.

### *In vitro* release kinetics

The employed poly(lactic) acid to poly(glycolic) acid ratio together with their molecular weights were selected to follow a certain degradation pattern which controls the release kinetics so a constant release of auranofin was achieved in the first 6 h of the experiment. The release kinetics from PLGA NPs is mainly based on PLGA degradation via hydrolysis of its ester linkages in the presence of water. In that case, the typical release profile from PLGA NPs should consist on a zero order phase. However, there are other effects influencing the release kinetics, such as surface diffusion, bulk diffusion, polymer molecular weight variation and erosion of the NPs. All of them contribute to a complex process that is very difficult to reproduce in an individual equation. In our particular case, the auranofin release data showed in Fig. 1b can be fitted to a near-zero order kinetic models following the equation:


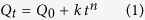


Being *Q*_*t*_ the percentage of auranofin released at time t, *Q*_*0*_ the initial amount of auranofin in the solution (normally *Q*_*0*_ = 0), *k* is the release constant, and *n* is the factor that is 1 in pure zero-order release (degradation of polymeric matrix) and 0.5 in Higuchi-type release (diffusion of drug molecules).

The parameters of the kinetic fitting, shown in Fig. 1b, indicate that our release process is not a pure zero release (degradation) neither a pure Higuchi release (diffusion), but somewhere in the middle (*n* = 0.81), a near-zero order kinetic release.

### *In vitro* bactericidal activity of auranofin-PLGA NPs

**Figure 2:**
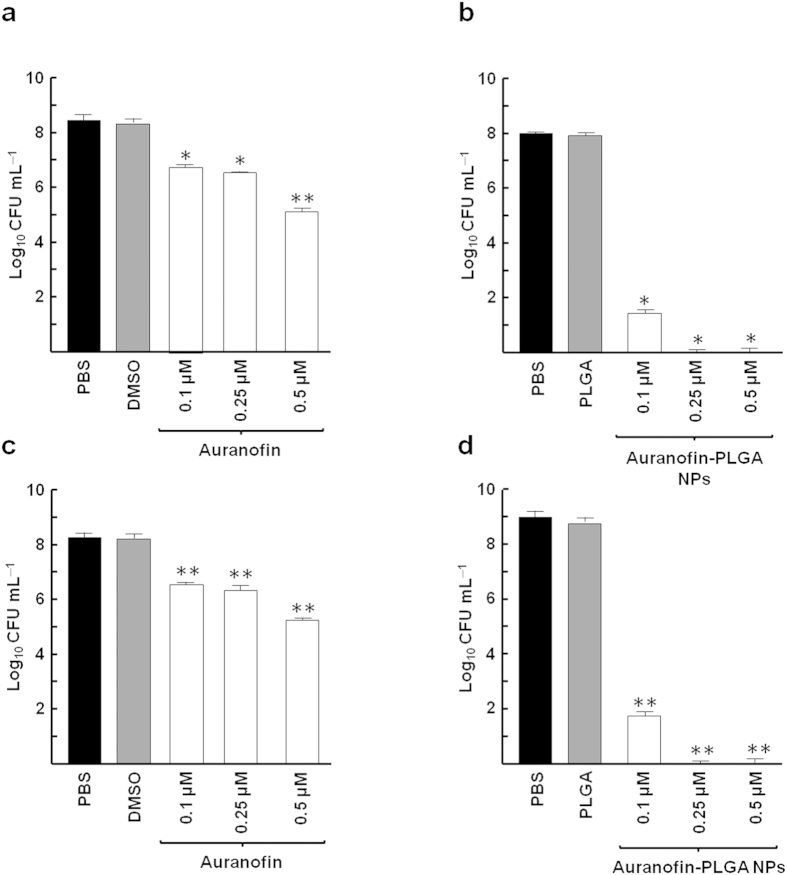
Bactericidal effects of auranofin and auranofin-PLGA NPs against pneumococcal strains. Exponentially growing cultures of R6 strain (**a**,**b**) and 48 strain (**c**,**d**) were incubated in the absence or in the presence of auranofin alone (**a**,**c**) or auranofin-NPs (**b**,**d**). Controls with PBS buffer, DMSO or PLGA without auranofin were included. Viable cells were determined on blood agar plates after 6 h of incubation at 37 °C. Data are means of four independent experiments. Error bars represent standard deviations, and asterisks indicate that results are statistically significant compared to the control in the absence of auranofin (one-way ANOVA with a *post hoc* Dunnet test; *P < 0.01; **P < 0.001).

To compare the bactericidal activity of auranofin alone or auranofin-loaded NPs, we initially chose the noncapsulated strain *S. pneumoniae* R6. It is important to notice that pneumococcal strains show autolysis after several hours of stationary phase of growth. Thus, the incubation time was adjusted to take into account the release kinetics of auranofin and bacterial autolysis in the conditions tested. The compounds were added to a bacterial suspension at an OD_550_ of 0.6 at three different concentrations (0.1, 0.25 and 0.5 μM), and cell optical density was followed throughout the growth curve. Viable cell counting demonstrated that auranofin alone was effective as an anti-pneumococcal agent since 6 h of incubation at 0.5 μM decreased bacterial population for about three log units (Fig. 2a), whereas cultures treated with auranofin-NPs were virtually sterilized at concentrations of 0.25 or 0.5 μM (Fig. 2b). Afterwards, the bactericidal activity of auranofin-loaded NPs was also tested using encapsulated pneumococcal isolates, namely, strains D39, 48 and 69 (Table 1). As in the previous assay, free auranofin caused a decrease on viability of almost three logs on the multiresistant 48 strain at 0.5 μM (Fig. 2c), whereas auranofin-NPs also led to total killing of the culture even at 0.25 μM (Fig. 2d). Comparable results were obtained with the D39 strain (serotype 2) and the multiresistant 69 strain (serotype 19F) (Table 2). To confirm that the bactericidal effect was produced by the auranofin released from NPs rather than from the NPs themselves, unloaded PLGA NPs were always tested in all the experiments, demonstrating that the nanocarrier did not alter the bacterial population.

Next, we tested the efficacy of auranofin, alone or loaded in NPs, against two strains of *S. pyogenes*, the type strain and SF370, the latter being a typical biofilm-forming strain. As shown in Table 2, the bactericidal effect in these strains did not reach the same levels than those found against pneumococcal strains. In fact, free auranofin did not cause any effect on the survival of the *S. pyogenes* type strain, even at the maximum concentration tested (0.5 μM) but provoked a 90%-drop in the viability of the SF370 strain. It is worth noting, however, that auranofin-NPs effectively killed both *S. pyogenes* strains, with a decrease of 1- and 2-logs on type strain and SF370 strain, respectively, confirming the increased efficiency of auranofin when was loaded into NPs, compared with auranofin alone.

**Figure 3:**
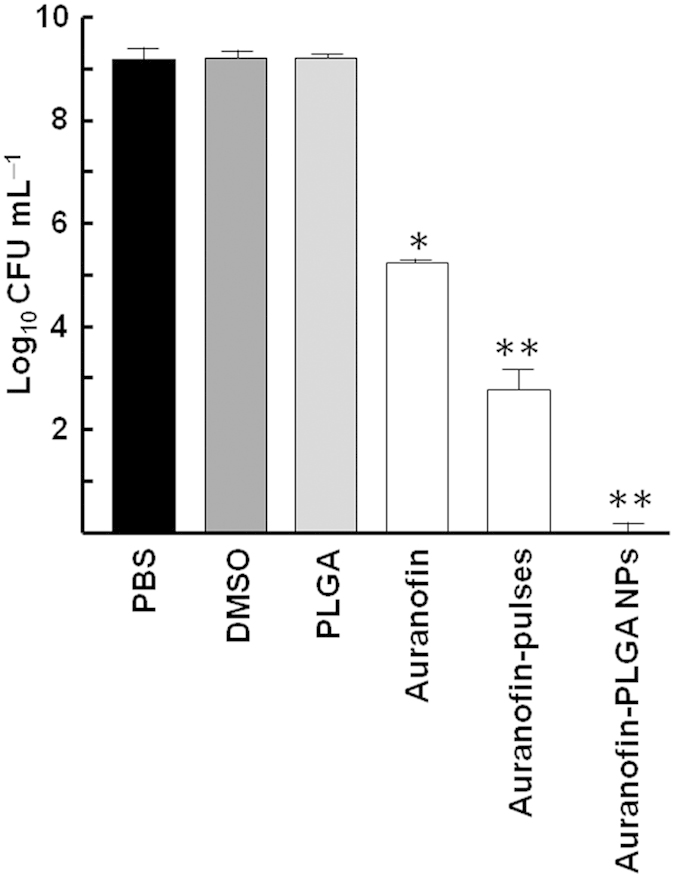
Bactericidal effects of auranofin pulses against *S. pneumoniae* R6. Exponentially growing cultures were incubated in the presence of a final concentration of 0.5 μM of auranofin added in three different conditions: as a single dose at the beginning of the experiment (auranofin), by 1 h pulses for 6 h (auranofin-pulses), or loaded into PLGA NPs (auranofin-PLGA NPs). Controls with PBS buffer, DMSO or PLGA without auranofin were also included. Viable cells were determined on blood agar plates after 6 h of incubation at 37 °C. Data are means of three independent experiments. Error bars represent standard deviations, and asterisks indicate that results are statistically significant compared to the control in the absence of auranofin (one-way ANOVA with a *post hoc* Dunnet test; *P < 0.01; **P < 0.001).

One of the reasons to explain the greater efficacy of auranofin loaded into PLGA NPs against the streptococcal pathogens could be due to a better stability of the encapsulated drug and, thus, to the protection against a rapid enzymatic and/or hydrolytic degradation^26^. To investigate this issue we performed bactericidal assays against *S. pneumoniae* R6 adding auranofin pulses each hour to simulate the slow release that takes place from the auranofin-NPs. Adjustment to a final concentration of 0.5 μM of auranofin by 6 pulses decreased bacterial population 6 logs after 6 h of treatment, while the same concentration of auranofin added at once at the beginning of the experiment caused a viability drop of almost 4 logs (Fig. 3). These results strongly suggest that treatments with repeated doses of auranofin could be more effective against pneumococcal infections than a high single dose. In other words, encapsulation of auranofin in PLGA NPs allows a good delivery system since a sustained release of the drug increases the efficiency of the treatment.

### Lethal activity of auranofin-PLGA NPs on bacterial biofilm model

**Figure 4:**
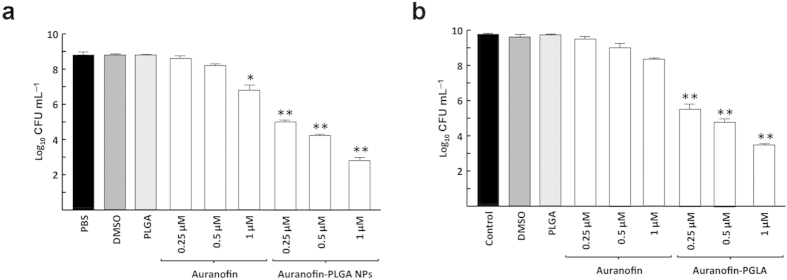
Streptococcal biofilms treated with auranofin or auranofin-PLGA NPs. (**a**) Cells of *S. pneumoniae* P046 strain, grown as biofilm, were treated with 0.25, 0.5 and 1 μM of auranofin and auranofin-PLGA NPs for 6 h at 37 °C. (**b**) Cells of *S. pyogenes* SF370 strain, grown as biofilm, were treated with 0.25, 0.5 and 1 μM of auranofin and auranofin-PLGA NPs for 6 h at 37 °C. Controls included biofilms incubated for 6 h in PBS buffer, DMSO or PLGA NPs. Viable cells were determined on blood agar plates. Data represent the mean of three independent experiments. Error bars represent standard deviations, and asterisks indicate that results are statistically significant compared to the control in the absence of auranofin (one-way ANOVA with a post hoc Dunnet test; *P < 0.01; **P < 0.001).

Free auranofin and auranofin-loaded in NPs were firstly assayed on *S. pneumoniae* P046 strain at concentrations of 0.25, 0.5 and 1 μM. This biofilm-prone strain does not autolyse after long periods of incubation either at 30 °C or 37 °C, due to its deficiency in LytA and LytC autolysins. A positive effect of any drug against bacterial biofilm is evidenced by the decrease of biofilm formation due to the dispersion of the cells and, most importantly, by the lethal action of the drug on the remaining cells. Consequently, the bactericidal effect on the P046 biofilm was checked after 6 h of the corresponding treatment and results clearly demonstrated that the drug charged in NPs killed more efficiently *S. pneumoniae* biofilm-grown cells than free auranofin. For instance, auranofin-NPs killed about 4 logs of the bacterial population at 0.25 μM (more than 99.9% mortality), whereas auranofin alone was virtually ineffective at the same concentration, being necessary at least 1 μM of compound to achieve 90% mortality (Fig. 4a). In addition, we checked the same treatments on *S. pyogenes* SF370 strain, grown as biofilm, at the same concentrations used before. The results in this case were very similar than the preceding experiment, as auranofin-NPs at a concentration of 0.25 μM were capable to kill the same 4 logs of bacterial cells. It is worth noting a dramatically different behavior in lethal activity between free and encapsulated auranofin when compared planktonic and biofilm-grown cultures of both pathogens, *S. pneumoniae* and *S. pyogenes*. As shown in Fig. 4 and Table 2, free auranofin displayed higher bactericidal effect on planktonic than on biofilm cells of *S. pyogenes* SF370 strain (about 1 and 0.5 log decrease, respectively, at 0.5 μM). In contrast, auranofin-loaded PLGA NPs were clearly more lethal against biofilm of both bacteria than the same cells grown planktonically (>4 and 2 log decrease, respectively, at 0.5 μM for *S. pyogenes*).

**Figure 5:**
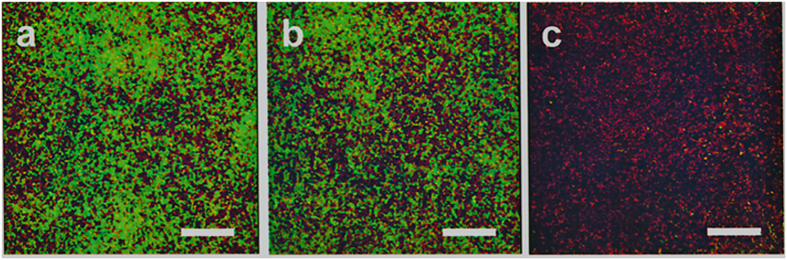
CLSM of pneumococcal biofilms treated with auranofin-PLGA NPs. Cells of *S. pneumoniae* P046 strain, grown as biofilm, were incubated with PBS (**a**), or with1 μM of PLGA NPs (**b**), or treated with1 μM of auranofin-PLGA NPs (**c**) for 6 h at 37 °C. Afterwards, cells in the biofilms were stained with the BacLight LIVE/DEAD kit to reveal viable (green fluorescence) and non-viable (red fluorescence) bacteria. Horizontal three-dimensional reconstructions (*x*–*y* plane) are shown. Bars, 25 μm.

Once demonstrated the powerful killing effect of auranofin-NPs against two streptococcal bacteria grown as biofilm, we analyzed by confocal laser scanning microscopy (CLSM) the fine structure of the typical mesh that characterizes this bacterial growth to detect the cell dispersion capacity due to the activity of auranofin-NPs. This analysis was made on P046 pneumococcal strain and CLSM images of the biofilm in PBS and the corresponding to a control containing unloaded PLGA NPs presented similar cell density and percentage of living bacteria (Fig. 5a,b). In contrast, the biofilm treated with auranofin-NPs evidenced the great loss of remaining pneumococcal cells (Fig. 5c), which fully confirmed the lethal action of auranofin when is loaded in PLGA NPs.

### Antibacterial activity in an animal model

**Figure 6:**
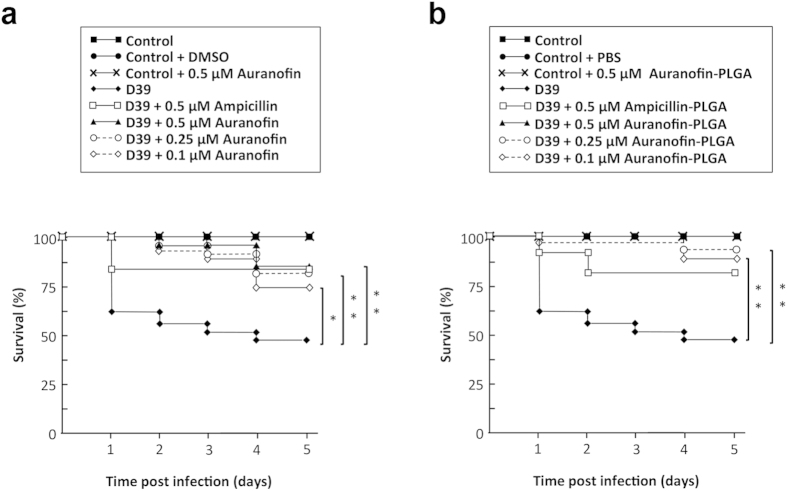
Rescue of zebrafish embryos from pneumococcal infection by auranofin or auranofin-PLGA NPs. Survival of embryos infected with *S. pneumoniae* D39 strain and treated (or not) with different concentrations of auranofin or auranofin-PLGA NPs (n= 24 to 36 embryos per condition) is shown. Controls with DMSO, auranofin, ampicillin alone and loaded into PLGA NPs were also included. Embryos were monitored for survival over a period of 5 days and results were plotted as Kaplan–Meier survival curves. Survival curves were compared with the log-rank (Mantel–Cox) and Gehan–Breslow–Wilcoxon tests (*P < 0.01; **P < 0.001).

**Figure 7:**
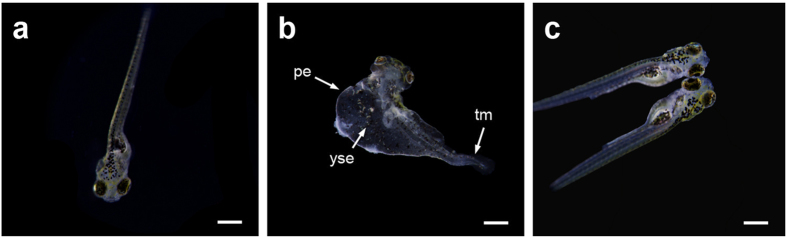
Microscopic images of zebrafish embryos infected with pneumococci and treated with auranofin-PLGA NPs. (**a**) Uninfected embryo group. (**b**) Embryo group infected with *S. pneumoniae* D39 strain without treatment. (**c**) Infected embryo group and treated with 0.5 μM auranofin-PLGA NPs. Images were taken after 5 days post infection. Abbreviations: pe, pericardial edema; tm: tail malformation, and yse, yolk sac edema. Bars, 500 μm.

To validate in an animal model of infection the *in vitro* bactericidal results of auranofin-NPs, we employed a zebrafish embryo model, which has been recently set up for *S. pneumoniae* and *S. pyogenes*^35^. Control experiments using the pneumococcal strain D39 showed that a bacterial challenge with about 2.5 × 10^8^ CFU mL^−1^ killed 50% of the embryos in 4−5 days, when administered by immersion in E3 medium. Therefore, at 48 h post fecundation, zebrafish embryos were brought in contact with the pathogen. Eight hours after bacterial challenge, embryos were extensively washed with the same medium and treated with a single dose of free auranofin or auranofin-NPs at concentrations ranging from 0.1 to 0.5 μM per embryo. Heat-killed (10 min at 65 °C) D39 strain cells were used as negative control. Rescue of the embryos infected and treated with auranofin-NPs varied depending on the concentration used, *i.e.,* 100% survival (46/46) when treated with 0.5 μM, 93.5% (43/46) with 0.25 μM, and 89.1% (41/46) with 0.1 μM. These results indicated the efficiency of auranofin to protect embryos from pneumococcal infection and also that treatment with the auranofin-NPs resulted in about 15% greater protection (*P* < 0.01) than with the drug alone, *e.g.* 73.9% of embryos (34/46) survived when treated with free auranofin and 89.1% with auranofin-NPs, both at the same concentration (0.1 μM) (Fig. 6). The effectiveness of auranofin on embryo rescue was compared with that of a conventional antibiotic like ampicillin, which belongs to the β-lactam family. When both drugs were added alone at 0.5 μM the zebrafish embryo survival was almost the same after 5 days post infection (84.6%) (Fig. 6a). However, treatments with the same concentration of ampicillin loaded into PLGA NPs rescued a similar rate of embryos (38/46 that means 82.6%), whereas auranofin-NPs achieved 100% of survival at the same concentration of 0.5 μM (Fig. 6b). To visualize the protection effect of auranofin-NPs, embryos from all groups of experiments were monitored over time for any morphological change. Embryos were examined using a stereomicroscope and it was found that embryos infected with *S. pneumoniae* D39 had a much shorter and curved tail and several deformities, mainly in pericardial cavity and yolk sac (Fig. 7b) compared to the corresponding uninfected controls (Fig. 7a). However, infected embryos and treated with 0.5 μM of auranofin-NPs were fully protected and did not show the typical deformities provoked by pneumococcal infection (Fig. 7c).

## Discussion

Infections caused by multiresistant bacteria are of important concern in terms of global health as they often promote lethal pathologies. It is becoming clear the particular relevance of biofilm-derived infections since after treatment with high concentrations of antibiotics most of the bacteria survived in such biofilms, even when dead bacterial cells covered the surface^7^. Disease treatments have been hampered mainly due to the lack of effective antibiotics; hence, the identification of new drugs with novel mechanisms of action has become an international priority^36^,^37^. Auranofin is a drug with a gold(I) center coordinated to a thiosugar and triethylphosphine residues, which has been recently repurposed as an efficient drug not only against pathogenic protozoa^12^,^13^ but also against several Gram-positive and Gram-negative bacteria, although the latter group was less susceptible to the drug than the former. In fact, MIC values for the most susceptible tested Gram-positive bacteria ranged between 0.12 and 0.5 μg mL^−1^, whereas these MICs increased to >16 μg mL^−1^ for some Gram-negative bacteria, like *Klebsiella pneumoniae, Pseudomonas aeruginosa* or *Acinetobacter baumannii*^15^,^16^. Although the detailed mechanism of the bactericidal effect of auranofin has not been elucidated, recent data strongly suggested that the thiol-redox homeostasis is the bacterial target in *M. tuberculosis*^16^. It was demonstrated that auranofin inhibits the bacterial thioredoxin reductase, a key protein in many Gram-positive bacteria for maintaining the thiol-redox balance and protecting against reactive oxidative species^15^.

Initially, we focused this study on the bactericidal effect of auranofin against two clinically relevant pathogens, *S. pneumoniae* and *S. pyogenes*. A summary of viability data, shown in Table 2, demonstrated that this drug has a potent bactericidal effect against all the pneumococcal strains tested, including the multiresistant ones, whereas it was rather inefficient against the *S. pyogenes* strains assayed. The value of the lethal concentration for pneumococci, 0.5 μM corresponds to 0.34 μg mL^−1^, is in the range of the MIC values reported for other Gram-positive bacteria as *S. aureus* and *Streptococcus epidermidis*^16^,^38^. After these promising results with the reprofiled auranofin against pneumococci, we looked for a method to improve and extend the bacteriolytic action of the drug, even in biofilm assays where bacterial population is more tolerant, or resistant, to antibiotic therapies. A typical solution to achieve this goal in the formulation of active compounds is loading of the desired drug in an appropriate vehicle or NP.

Polymers have been employed by the pharmaceutical industry for more than 40 years, evolving from resorbable sutures and orthopaedic implants to multifunctional NPs able of targeting and controlled release of therapeutics. In this sense, the use of polymeric NPs for developing safer and more effective medicines, so-called nanomedicines, has substantially influenced the pharmaceutical and biotechnological industries. Among others, biodegradable PLGA NPs have been widely employed for a variety of biological applications thanks to their reliable methods of preparation, the biocompatibility (FDA-approved), biodegradability^39^,^40^, the capacity to encapsulate and protect drugs from degradation^21^,^41^. Additionally, active molecule entrapping (or encapsulating) within a delivery system provides a greater control of the pharmacokinetic behavior of the desired drug. This more efficient use of pharmaceutical compounds may diminish some of the drawbacks and supplies the basis for shortening the current time required by classical treatments. This might be of capital importance when dealing with antibiotics, because it could help to avoid the resistance of bacteria to antibiotics, which is one of the main concerns in the treatment of infections nowadays. There are many examples in the literature where the encapsulation of antibiotics into PLGA NPs increases their efficiency. For example, azithromycin loaded PLGA NPs were more active than azithromycin solution against *Salmonella typhi*^42^. More recently it has been described that clarithromicine-PLGA NPs are more effective than untreated clarithromicine against *E. coli, H. influenzae, S. aureus* and *S. pneumoniae*^43^. Given the advantages showed by PLGA NPs and taking into account the improved efficacy of antibiotics when loaded in this delivery system, we prepared auranofin-PLGA NPs with a protocol that allowed the reproducible formation of NPs exhibiting diameters below 100 nm and low polydispersity indexes, which is indicative of a homogeneous size distribution.

Once the loading and sustained release of auranofin from PLGA NPs was confirmed, their potential benefits for infection treatments were evaluated. Specifically, preparation of auranofin-PLGA allowed the comparison of its lethal activity against *S. pneumoniae* and *S. pyogenes* with that of auranofin alone using three kinds of experiments as model systems: i) *in vitro* study using several pneumococcal strains, including relevant multiresistant ones; ii) biofilm assay on appropriate strains, like *S. pneumoniae* P046 and *S. pyogenes* SF370; iii) *in vivo* animal model, like zebrafish embryos infected with *S. pneumoniae*. All these approaches confirmed the improved killing activity of auranofin-NPs over the free auranofin with particular emphasis on the biofilm approach, since the potency of auranofin loaded into PLGA to disperse and kill bacterial population of *S. pneumoniae* and *S. pyogenes* biofilms exceeded about 4 logs the lethality found with the drug alone against both pathogens. This promising result could be especially relevant in foreseeing a future treatment of biofilm-based infections that are particularly recalcitrant to classical antibiotics^44^. An additional argument to count on clinical application of this kind of NPs comes from the successful validation of *in vitro* assays with the protection to infected zebrafish embryos, which highlights the benefits of NPs as drug delivery system to combat streptococcal infections.

## Methods

### NP preparation

The nanoprecipitation method for the formation of auranofin-encapsulated PLGA NPs was carried out according to a previously described procedure^45^. Briefly, 10−15 mg of auranofin [Sigma-Aldrich, >98% High Performance Liquid Chromatography (HPLC)] were dissolved in 20 mL of a mixture of dichloromethane:acetone (0.5:19.5), and then 200 mg of PLGA (Aldrich, 50:50, Mw 7,000–17,000) were added and subsequently sonicated for 2 min (solution 1). Separately, 56 mg of Pluronic F-68 (Sigma) were dissolved in 40 mL of water (0.14% w v^−1^) in a 100 mL round bottom flask under moderate magnetic stirring (solution 2). Solution 1 was placed on a syringe dispenser to be then transferred dropwise (0.25 mL min^−1^) over solution 2 under moderate magnetic stirring. The NPs were stirred for 2 h, and the remaining organic solvent was removed in a rotary evaporator at reduced pressure for 2 h. The NPs were then centrifuged at 6,600 rpm in a 7700 RCF rotor at 4 °C for 15 min, washed twice with distilled water and centrifuged again. Then, the NPs were suspended in a small amount of water, flash frozen in liquid nitrogen and freeze-dried. The same process was followed without auranofin for the synthesis of unloaded PLGA NPs.

### Morphology of the produced NPs

The spherical morphology of the produced NPs was evaluated using SEM on a JEOL JSM 6335F (Electron Microscopy Centre, UCM). The samples underwent Au metallization prior to observation.

### Determination of particle sizes and polydispersities

The average hydrodynamic size and the polydispersity index were measured with a DLS apparatus (Zetasizer NanoZS, Malvern Instruments) equipped with a 633 nm laser at 25 °C. Samples from the prepared suspensions with ultra-purified water (about 0.1 mg mL^−1^) were placed in the measurement cell.

### Drug loading and encapsulation efficiency

Auranofin was quantified measuring Au in an acid-digested sample by Inductively Coupled Plasma Atomic Emission Spectroscopy (ICP-AES, Varian Vista AX Pro) through the Au emission line at 267.6 nm. Briefly, 10 mg of auranofin-loaded NPs were precisely weighed and placed in a reactor Teflon-jacketed steel case. Then, 10–20 mL of concentrated hydrofluoric/nitric acid (1:1) (Panreac) were gently added and moderately heated until complete digestion for 48 h. Finally, the clear yellow solution was taken to 10 mL volumetric flask and measured. The achieved drug loading was 1.8–6.2 mg of auranofin per gram of NPs in all synthesis.

### *In vitro* release of auranofin from PLGA NPs

*In vitro* release studies were carried out dispersing 10 mg of loaded PLGA NPs in 0.5 mL of fresh phosphate-buffered saline (PBS) solution (137 mM NaCl, 2.7 mM KCl, 10 mM Na_2_HPO_4_, and 1.8 mM KH_2_PO_4_ [adjusted to pH 7.4]) and placed on a Transwell permeable support with a 0.4 μm (average pore size) polycarbonate membrane. The well was filled with 1.0 mL of PBS pH 7.4 and the suspension was orbitally shaken at 100 rpm at 37 °C during all the experiment. Every hour, 1 mL of sample was removed from the Transwell plate and replaced with fresh PBS. The separation and quantification was carried out by HPLC equipped with UV-Vis detector, measuring the absorbance of 2-nitro-5-thiobenzoate (NTB, λ_max_ 409 nm). NTB is produced when thiol groups react with 5,5′-dithiobis-(2-nitrobenzoic acid) (DTNB) cleaving the disulphide bond to obtain NTB in water at neutral pH. The samples containing auranofin were treated with KI to break the gold-thiol bond^46^. With this purpose 250 μL of sample with 50 μL KI 2.0 M were placed in an ultrasound bath at 50 °C for 20 min, then immediately ice-cooled, according to the standard protocol provided by Sigma. An HPLC (Waters Alliance 2695) separation module (Milford, Massachusetts, USA) based on a isocratic mobile phase of ACN:MeOH:Water (3:3:94, v:v:v) at a flow rate of 1 mL min^−1^ was used to separate and quantify the NTB signal with a 4.2 min of retention time. The system was equipped with a variable wavelength diode array detector Water 2996 at 354 nm. A Waters X-Terra RP-18 reverse phase column (4.6 × 250 mm, 5 μm) was employed at 40 °C and 10–50 μL injection volume was selected upon the case. A scheme describing the reactions, the protocol for derivatization of auranofin, and the 3D chromatogram is shown in Fig. S1.

### Bactericidal assays

Bactericidal effects of the auranofin-loaded NPs were monitored by following the optical density at 550 nm (OD_550_) for 6 h and the viable cells at this time point. Briefly, *S. pneumoniae* and *S. pyogenes* strains (Table 1) were grown in C medium^47^ and Todd-Hewitt medium, respectively, supplemented with yeast extract (0.8 mg mL^−1^; Difco Laboratories) at 37 °C without shaking. Once the bacteria reached the exponential phase of growth (OD_550_ ≈0.3), the cultures were centrifuged and washed twice with PBS, and the final OD_550_ was adjusted to ≈0.6 with PBS. Resuspended bacteria were then transferred into plastic tubes, and different amounts of auranofin-PLGA NPs (suspended in PBS) were added to reach a final concentration of 0.1, 0.25, or 0.5 μM auranofin. Control samples with unloaded PLGA NPs or auranofin alone at the same concentrations were always run in parallel. Additional controls with PBS and dimethylsulfoxide (DMSO), used to solubilize NPs and auranofin, respectively, were also included. All samples were incubated at 37 °C for 6 h, and the OD_550_ and viable cells were determined at selected time points. Viable cell counting was carried out in tryptose blood agar base plates (Difco Laboratories) supplemented with 5% defibrinated sheep blood. For each sample, a 10-fold dilution series was prepared in PBS, and 10 μL of each dilution were plated. Colonies were counted after overnight incubation at 37 °C.

### Biofilm assay

Pneumococcal biofilms were produced using *S. pneumoniae* strain P046, a double *lytA lytC* mutant^48^, a derivative from the unencapsulated laboratory strain R6^49^. The optimal conditions for biofilm formation on polystyrene plates have been described elsewhere^50^. In short, all biofilms were formed in Costar 3595 96-well polystyrene microtiter plates (Corning, New York, USA). Cells were grown in C medium supplemented with yeast extract (0.8 mg mL^−1^) to an optical density of 0.5–0.6 at OD_595_, sedimented by centrifugation, resuspended in an equal volume of C medium, diluted, and portions of 200 μL containing 2.2 × 10^4^ CFU were dispensed into each well. After 16 h of incubation at 34 °C, the planktonic cultures were removed and the resulting biofilms were washed twice with PBS and then treated for additional 6 h at 37 °C with various concentrations of auranofin or auranofin-NPs. Controls with DMSO or unloaded NPs were also assayed. Afterwards, the supernatants were removed again, washed twice with PBS and 10-fold dilution series were prepared in PBS. 10 μL of each dilution were plated in blood agar plates and colonies were counted after overnight incubation at 37 °C. The *S. pyogenes* biofilm was produced using SF370 strain, as previously described^51^. Briefly, an overnight culture of *S. pyogenes* was diluted 1:20 with fresh Todd-Hewitt medium supplemented with yeast extract (0.8 mg mL^−1^) and grown statically in polystyrene microtiter plates for 24 h at 37 °C. After incubation, the same protocol was followed as described above for the pneumococcal biofilms.

### Microscopy observation of biofilms

To observe biofilms by CLSM, *S. pneumoniae* P046 strain was grown on glass-bottomed WillCo-dishes (WillCo Wells) for 16 h at 34 °C. Following incubation, the culture medium was removed and the biofilm rinsed with PBS to remove non-adherent bacteria and then treated for additional 6 h at 37 °C with 1 μM of auranofin-NPs. Control biofilms with PBS or unloaded PLGA NPs were also assayed. Afterwards, the supernatants were removed again and then stained with the LIVE/DEAD *Bac*Light bacterial viability kit L-13152 (Invitrogen-Molecular Probes) for monitoring the viability of bacterial populations. Cells with a compromised membrane — considered dead or dying— stain red, whereas those with an intact membrane stain green^48^. The biofilms were observed at ×63 magnification using a Leica TCS-SP2-AOBS-UV CLSM. The excitation/emission maxima were around 488/500–546 nm. Images were analyzed using LCS software (Leica). Projections were obtained through the *x*–*y* plane (individual scans at 0.5-μm intervals).

### Zebrafish embryo infection assay

The pneumococcal infection model was based on a method described previously^35^, using wild-type zebrafish embryos (*Danio rerio*) obtained from ZFBioLabs (Tres Cantos, Spain). Briefly, embryos were dechorionated at 24 h post fecundation by treatment with pronase (2 mg mL^−1^) for 1 min, distributed in 96-well plates and incubated in 100 μL of E3 medium. At 48 h post fecundation, embryos were infected with 100 μL of a 2.5 × 10^8^ colony-forming units (CFU) mL^−1^ suspension of *S. pneumoniae* strain D39. Bacterial inoculation titers were calculated by serial dilution and plating onto tryptose blood agar plates for each experiment. Seven hours post infection, infected embryos were extensively washed with E3 medium to remove the bacteria and 100 μL of the same autoclaved fresh medium supplemented with auranofin (0.1, 0.25 or 0.5 μM) or auranofin-loaded NPs (containing the same concentration of the drug) were added. Non-infected controls without treatment or treated either with DMSO, auranofin, or unloaded PLGA NPs were washed in the same way. Embryos were incubated at 27.5 °C under sterile conditions and mortality was followed in all samples for 5 days, changing fresh E3 medium every day. Zebrafish embryos were considered dead when coagulation was observed as well as absence of heartbeat during 15 seconds observation. Each experiment was repeated at least 3 times, and 24 to 36 embryos were used per condition and experiment.

### Ethics statement

All experiments with zebrafish embryos were performed under strict accordance to the new EU Directive 2010/63/EU on the protection of animals. Signs of infection were monitored three times daily throughout the experimental time course. Moribund embryos were anesthetized by immersion in tricaine (MS222) (Sigma-Aldrich) at 200 mg mL^−1^, followed by immobilization by submersion in ice water (5 parts ice/1 part water, 0–4 °C) for at least 20 min to ensure death by hypoxia. Approval for these experimental protocols was granted by the Spanish Royal Decree 53/2013 laying down basic standards for the protection of animals used in animal experiments and other scientific purposes.

### Imaging analysis

Live embryos (5 days post infection) were treated to ensure death as described above, mounted in 3% methylcellulose in depression slides and photographed under an Olympus SZX16 stereoscope with a QImaging MicroPublisher 5.0 RVT camera. Images were processed with NIH ImageJ. For all the experiments described, the images shown are representative of the effects observed in at least 90% of the individuals.

### Statistical analysis

All *in vitro* results are representative of data obtained from repeated independent experiments, and each value represents the mean standard deviations for 3 to 4 replicates. Statistical analysis was performed by using the two-tailed Student’s t test (for two groups), whereas analysis of variance (ANOVA) was chosen for multiple comparisons. For all *in vivo* data, the log-rank (Mantel-Cox) and Gehan-Breslow-Wilcoxon tests were used to draw, analyze and compare the survival curves. GraphPad InStat version 5.0 (GraphPad Software, San Diego, CA) was used for statistical analysis.

## Additional Information

**How to cite this article**: Díez-Martínez, R. *et al*. Auranofin-loaded nanoparticles as a new therapeutic tool to fight streptococcal infections. *Sci. Rep.* **6**, 19525; doi: 10.1038/srep19525 (2016).

## Supplementary Material

Supplementary Information

## Figures and Tables

**Table 1:** Bacterial strains used in this study.

Strain	Relevant description	Source or reference
*S. pneumoniae*		
R6	D39 derivative; non-encapsulated	49
D39	Serotype 2	52
48	Serotype 23F; penicillin MIC^a^ = 16 mg L^−1^; erythromycin MIC^a^ > 128 mg L^−1^; ciprofloxacin MIC^a^ = 1 mg L^−1^; levofloxacin MIC^a^ = 1 mg L^−1^; chloramphenicol MIC^a^ = 4 mg L^−1^; tetracycline MIC^a^ > 64 mg L^−1^	53
69	Serotype 19F; penicillin MIC^a^ = 2 mg L^−1^; erythromycin MIC^a^ = 16 mg L^−1^; levofloxacin MIC^a^ = 1 mg L^−1^; chloramphenicol MIC^a^ = 4 mg L^−1^; tetracycline MIC^a^ = 64 mg L^−1^	53
P046	*lytAlytC* mutant	48
*S. pyogenes*		
Type strain		CECT^b^ 985
SF370	Serotype M1; biofilm-forming strain	CECT^b^ 5109

^a^MIC, Minimum Inhibitory Concentration.

^b^CECT, Colección Española de Cultivos Tipo.

**Table 2:** Bacterial viability after treatment with auranofin or auranofin-PLGA NPs^a^.

Organism (serotype)	Viability after 6 h of treatment with^b^:							
	Auranofin (μM)				PLGA-auranofin (μM)			
	DMSO	0.1	0.25	0.5	PLGA	0.1	0.25	0.5
*S. pneumoniae*								
R6 (none)	−	+	+	+++	−	++++	*	*
D39 (2)	−	++	++	+++	−	++++	++++	*
48 (23F)	−	+	+	+++	−	++++	*	*
69 (19F)	−	++	++	+++	−	++++	++++	*
*S. pyogenes*								
Type strain	−	−	−	−	−	−	−	+
SF370 (M1)	−	−	−	+	−	+	++	++

^a^Bacteria were incubated at 37 °C in PBS (OD_550_ ≈ 0.6) with the indicated compound at 0.1, 0.25 or 0.5 μM. Viability was determined after 6 h of incubation.

^b^−, no effect; +, decrease of 1 log in viable cells;++, decrease of 2 logs in viable cells; +++, decrease of 3 logs in viable cells; ++++, decrease of ≥4 logs in viable cells; *, <10 CFU mL^−1^.
