# Supplementary material for: Auranofin-loaded nanoparticles as a new therapeutic tool to fight streptococcal infections
Source: Sci Rep. 2016 Jan 18;6:19525. doi: 10.1038/srep19525 (PMC4726118; doi:10.1038/srep19525)
Supplement: Supplementary Information [file srep19525-s1.pdf]

Supplementary material: Figure S1

Auranofin-loaded nanoparticles as a new therapeutic tool to fight streptococcal infections

Roberto Díez-Martínez, Esther García-Fernández, Miguel Manzano, Ángel Martínez, Mirian Domenech, María Vallet-Regí & Pedro García

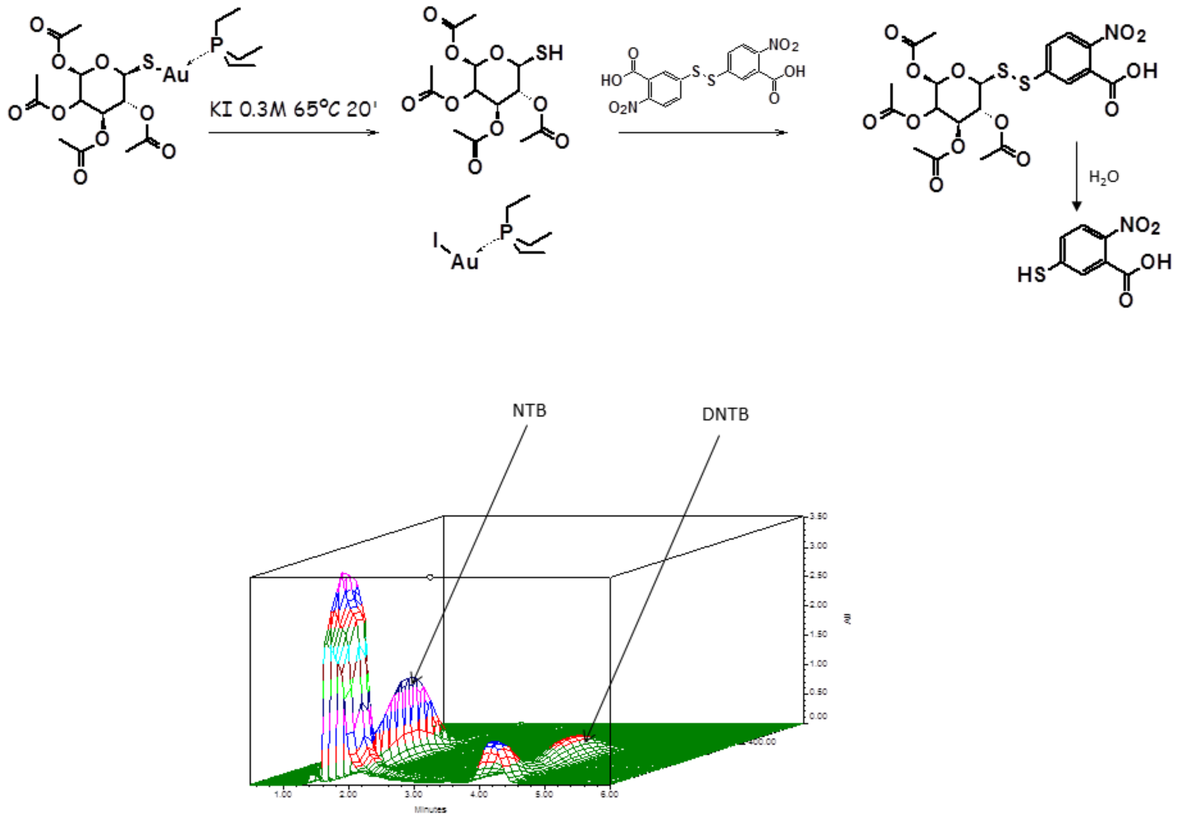

Figure S1. Derivatization of auranofin and HPLC measurements. Reactions scheme describing the protocol for derivatization of auranofin (top), and 3D chromatogram of the HPLC measurement of auranofin derivates. NTB, 2-nitro-5-thiobenzoate; DNTB, 5,5’-dithiobis-(2-nitrobenzoic acid).
